# Combined Linkage and Association Mapping Reveals QTL and Candidate Genes for Plant and Ear Height in Maize

**DOI:** 10.3389/fpls.2016.00833

**Published:** 2016-06-15

**Authors:** Xiaopeng Li, Zijian Zhou, Junqiang Ding, Yabin Wu, Bo Zhou, Ruixia Wang, Jinliang Ma, Shiwei Wang, Xuecai Zhang, Zongliang Xia, Jiafa Chen, Jianyu Wu

**Affiliations:** College of Agronomy, Synergetic Innovation Center of Henan Grain Crops and National Key Laboratory of Wheat and Maize Crop Science, Henan Agricultural UniversityZhengzhou, China

**Keywords:** plant height, ear height, association mapping, quantitative trait locus, candidate gene, maize

## Abstract

Plant height (PH) and ear height (EH) are two very important agronomic traits related to the population density and lodging in maize. In order to better understand of the genetic basis of nature variation in PH and EH, two bi-parental populations and one genome-wide association study (GWAS) population were used to map quantitative trait loci (QTL) for both traits. Phenotypic data analysis revealed a wide normal distribution and high heritability for PH and EH in the three populations, which indicated that maize height is a highly polygenic trait. A total of 21 QTL for PH and EH in three common genomic regions (bin 1.05, 5.04/05, and 6.04/05) were identified by QTL mapping in the two bi-parental populations under multiple environments. Additionally, 41 single nucleotide polymorphisms (SNPs) were identified for PH and EH by GWAS, of which 29 SNPs were located in 19 unique candidate gene regions. Most of the candidate genes were related to plant growth and development. One QTL on Chromosome 1 was further verified in a near-isogenic line (NIL) population, and GWAS identified a C_2_H_2_ zinc finger family protein that maybe the candidate gene for this QTL. These results revealed that nature variation of PH and EH are strongly controlled by multiple genes with low effect and facilitated a better understanding of the underlying mechanism of height in maize.

## Introduction

Maize (*Zea mays* L.) is one of the most cultivated crops worldwide, owing to its versatility and wide adaptability, and serves as food, animal feed, and raw material for various industrial products. Plant density is considered an important factor to increase yield (Cardwell, 1982; Zhang et al., 2014). Plant height (PH) and ear height (EH) are two important traits that directly affect plant density (Cai et al., 2012). A better understanding of the genetic architecture of PH and EH could help breeders to develop varieties with high density tolerance and, consequently, increased yield.

The mapping of quantitative trait loci (QTL) based on bi-parental populations is an effective method for detecting the genetic variation of in crops. Molecular markers have been widely used in QTL mapping for various traits in maize, including maturity (Buckler et al., 2009), disease resistance (Zwonitzer et al., 2010; Chen et al., 2012; Ding et al., 2012), abiotic stress (Agrama and Moussa, 1996), plant morphology (Xu et al., 2009; Ding et al., 2015; Chen et al., 2016), and plant color (Chen et al., 2014). More than 280 QTL have been identified for PH and EH in maize (Cai et al., 2012); Sibov et al. (2003) detected four QTL for PH and five QTL for EH that explained 24.76 and 20.91% of the phenotypic variance, respectively, using an F_2:3_ population with 400 families; Tang et al. (2006) detected six QTL for PH, of which four QTL were located in the same genomic region of the QTL affecting average internode length using a recombinant inbred line (RIL) population and suggested that average internode length was the main contributor to PH in maize; and Wang et al. (2006) detected 127 QTL for PH based on the maize integrated QTL map and confirmed 40 of them using meta-analysis. Additionally, Peiffer et al. (2014) showed that PH in maize has a highly polygenic genetic architecture using nested-association mapping population. Therefore, multiple models of genetic architecture, differing in polygenicity and effect sizes, can plausibly explain population variation for height in maize. All the previous studies provided valuable information to determine the genetic basis of height in maize, but only few QTL were validated in different populations. Therefore, the underlying mechanism of plant development remains unclear.

The available single nucleotide polymorphisms (SNPs) (Elshire et al., 2011; Chia et al., 2012) have been widely used in genome-wide association study (GWAS) for discovering loci controlling various traits in maize, including leaf architecture (Tian et al., 2011), disease and insect resistance (Zila et al., 2014; Samayoa et al., 2015; Li et al., 2016; Mahuku et al., 2016), and other important agronomic traits (Yang et al., 2014). Compared with traditional QTL mapping, GWAS has higher resolution and allows the identification of genes for multiple traits in a single population (Breseghello and Sorrells, 2006; Yu and Buckler, 2006; Yan et al., 2011). However, population substructure and genotyping errors can result in high false positive rates (Andersen et al., 2005; Yu et al., 2006). Therefore, the traditional QTL mapping powerfully compares pairs of alleles with low resolution, whereas association analysis provides a high-resolution evaluation of numerous alleles with uneven statistical power (Wilson et al., 2004). In this study, we combined linkage mapping and GWAS to identify QTL for PH and EH and exploit the strengths and weaknesses of both approaches for discovering causal loci across the genome.

Previous studies have cloned several genes that strongly influence PH in maize, such as brachytic2 on Chromosome (Chr.) 1, which influences polar auxin transport (Multani et al., 2003; Xing et al., 2015); ZmGA3ox2 on Chr. 3 and dwaft3 on Chr. 9 related to gibberellin synthesis (Winkler and Helentjaris, 1995; Teng et al., 2013); and dwarf8 on Chr. 1 and dwarf9 on Chr. 5 involved in gibberellin signal transduction pathways (Thornsberry et al., 2001; Lawit et al., 2010). In maize, over 40 genes have been reported for PH that are related to different biosynthesis pathways such as hormone synthesis, transport, and signaling (Wang and Li, 2008; Peiffer et al., 2014). However, the underlying mechanism of plant development remains unclear. The identification of additional loci/genes related to PH in elite inbred maize lines might help to better understand the mechanism of height development and optimize selection in breeding programs.

Here, we evaluated two bi-parental populations and one GWAS population for PH and EH in different environments and used both linkage mapping and GWAS to identify and verify QTL and candidate genes associated with the natural variation of height in maize. Our objectives were to: (1) identify QTL that significantly affect PH and EH in the two bi-parental populations; (2) discover top SNPs and their candidate genes associated with PH and EH by GWAS; (3) verify QTL in a near-isogenic line (NIL) populations; and (4) discuss the function of candidate genes.

## Materials and methods

### Germplasm and phenotype evaluation

An F_2:3_ bi-parental population, consisting of 225 families were developed from a cross between Zheng58 and Chang7-2, which are the parental lines of Zhengdan958, one of the most popular maize hybrids in China with an annual acreage of 4.0 × 10^6^ ha, accounting for 20% of total maize production (Du et al., 2006). Parental line Chang7-2 has higher PH and EH than another parental line Zheng58. The population and their parents were evaluated in Zhengzhou (34°52′N 113°37′E), Jiyuan (35°34′N 112°5′E), and Xichang (27°32′N 102°10′E) in 2007 (07Zhengzhou, 07Jiyuan, 07Xichang) and Zhengzhou and Jiyuan in 20008 (08Zhengzhou, 08Jiyuan). Another bi-parental recombinant inbred line (RIL) population consisting of 250 lines was produced from a cross between BT-1 and N6. BT-1 is an inbred line reformed by tropical Asia material with relatively high PH and EH and high resistance to various diseases, whereas N6 is a Tangsipingtou inbred line with relatively low PH and EH and low resistance to diseases. The population and their parents were evaluated in Zhengzhou in 2007 and 2008 (07Zhengzhou, 08Zhengzhou), Xuchang (34°2′N 113°81′E) in 2009 (09Xuchang), and Zhengzhou in spring and summer of 2009 (09Zhengzhou1, 09Zhengzhou2).

NIL population was developed from a cross between the low-PH line Zheng58, and the donor line Chang7-2 through four cycles of advanced backcrosses and one generation of self-crossing using marker assisted selection for the target genomic region. Two NILs, one homozygous for the QTL of the donor parent (+) and another homozygous for the QTL of the recurrent parent (−) were evaluated in Zhengzhou and Wenxian (34°95′N 113°6′E) in summer 2009.

The GWAS population, consisting of 258 maize inbred lines including the parental inbred lines of the two bi-parental populations, from the heterotic populations Tangsipingtou and Reid and some tropical lines from International Maize and Wheat Improve Center (CIMMYT), was evaluated in Zhengzhou, Wenxian and Hainan (18°21′N 109°10′E) in 2015 (15Zhengzhou, 15Wenxian, and 15Hainan, respectively).

All the populations were arranged in a randomized complete block design with three replications for each location. Sixteen plants were planted in 4 m row plots with 0.67 m row spacing. Field management was performed according to the standard agronomic practices in each location. PH was measured from the soil level to the tip of main inflorescence, and EH was measured from the soil level to the node attachment of the primary ear. The final value of each plot was the mean value of each plant inside the plot.

### Phenotypic data analysis

Analysis of variance (ANOVA) of phenotype data was performed using the SPSS software (www.spss.com). The components of variance were estimated using a complete random effects model and broad-sense heritability was calculated as defined by Knapp et al. (1985). Best linear unbiased predictions (BLUPs) of the combined PH and EH were calculated by using a mixed linear model (lmer) in R software (R Core Team, 2015), which used replication, environments which combination location and years, and entries as a random effect. The BLUP value of each line was used for GWAS analysis. Histogram of phenotypic data was generated by using hist function in R software (R Core Team, 2015).

### Linkage mapping

The two bi-parental populations were genotyped by simple sequence repeat (SSR) markers. The linkage map of RIL population contained 207 polymorphic SSR markers had a total length of 1820.8 cM and an average distance of 11.7 cM between markers (Li et al., 2011). The F_2:3_ population was genotyped with 180 SSR markers, and the constructed map had a total length of 1987.7 cM and an average distance of 11.0 cM between markers (Ding et al., 2011).

Composite interval mapping (CIM) for QTL mapping was performed by Windows QTL Cartographer 2.5 (Zeng, 1994). The threshold value was set using 1000 random permutations (Churchill and Doerge, 1994). The proportion of phenotypic variation explained by a single QTL was determined by the square of the partial correlation coefficient (R^2^).

### GWAS and candidate gene annotation

A total of 955,650 SNPs were identified in the GWAS population using the genotyping-by-sequencing (GBS) method (Elshire et al., 2011; Glaubitz et al., 2014) which was conducted in Cornell University and the SNP flanking sequence and position information were available in “panzea” website (http://cbsusrv04.tc.cornell.edu/users/panzea/download.aspx?filegroupid=4). The SNPs was filtered with a missing value greater than 0.25 and a minor allele frequency (MAF) less than 0.05, resulting in 224,152 SNPs for future analysis. The principal component analysis (PCA), Kinship matrix and linkage disequilibrium (LD) between each pair of SNPs were conducted using TASSEL 5.0 software (Bradbury et al., 2007). The population structure was determined using an admixture ancestry model with correlated allele frequency in software STRUCTURE v2.3.3 (Pritchard et al., 2000). STRUCTURE was run four replicates for K (number of subpopulations) = 1–8, and two replicates for *K* = 9–12 with a run-length of 100,000 repetitions of Markov chain Monte Carlo following a burn-in period of 10,000 iterations. GWAS was conducted using a mixed linear model (MLM) that included BLUPs, markers, Kinship and PCA in TASSEL (Bradbury et al., 2007).

Candidate gene information were obtained from the MaizeGDB (http://www.maizegdb.org/) genome browser based on physical position of significant SNPs. Phytozome database (http://phytozome.jgi.doe.gov/pz/portal.html) were used to define relevant pathways and annotate possible functions of candidate genes.

### Meta-analysis

All QTL detected in the bi-parental populations and SNPs identified in the GWAS population were analyzed based on their physical position using R (R Core Team, 2015). The R code is provided in File S1.

## Results

### Phenotype analysis

Descriptive statistics for PH and EH in the three mapping populations are presented in Table 1. There were a wide variations were observed in each population, for example, the PH of combined environments in RIL population ranged from 105 to 273 cm, whereas in the GWAS population from 106 to 293 cm. The EH in the F_2:3_ population ranged from 38.8 to 123 cm, and from 21.7 to 147 cm in GWAS population. The mean of PH and EH in the F_2:3_ families were 183.6 and 73.7 cm, respectively. The frequency of phenotypic value in all three populations for PH and EH followed an approximately normal distribution (Figure S1). The genotypic variance ($\sigma_{\text{g}}^{2}$) and the genotype-by-environment variance ($\sigma_{\text{ge}}^{2}$) of PH and EH were significant in all three populations. Heritability for PH was 0.92 in the F_2:3_ population, 0.97 in the RIL population, and 0.91 in the GWAS population. Heritability for EH is similar to that for PH (Table 1). The high repeatability and heritability indicated that much of the phenotypic variance was genetically controlled in the populations and suitable for QTL mapping.

**Table 1:** **Mean and standard deviation (SD) values, variance components and heritability of plant height (PH) and ear height (EH) in the F_**2:3**_ population, the recombinant inbred line (RIL) population, and the genome-wide association study (GWAS) population**.

**Population**	**Trait**	**Environment**	**Mean ± SD^a^^,^^b^**	**Range^a^**	**Short Parent^a^**	**High Parent^a^**	**$\sigma_{\text{g}}^{2}$**	**$\sigma_{\text{ge}}^{2}$**	***H*^2^^c^**
F_2:3_	PH	07Zhengzhou	182.9±15.6	141.5–231.7	151.2	181.7	245.5		0.75
		07Jiyuan	193.6±17.3	153.4–248.9	154.8	176.6	271.3		0.72
		07Xichang	160.8±14.9	124.6–213.8	136.9	140.8	487.4^**^		0.95
		08Zhengzhou	179.8±16.8	112.1–233.5	152.5	171.6	632.3^**^		0.95
		08Jiyuan	197.8±17.7	150.1–249.3	166.5	197.9	855.1^**^		0.98
		Combined	183.6±20.8	112.1–249.3	152.4	173.7	1091.2^**^	343.4^**^	0.92
	EH	07Zhengzhou	73.5±9.8	47.7–108.6	54.7	88.1	97.2		0.75
		07Jiyuan	75.9±10.8	47.1–112.8	47.9	80.8	111.1		0.74
		07Xichang	60.1±9.3	38.8–94.3	43.2	60.1	186.8^**^		0.94
		08Zhengzhou	72.2±10.4	42.6–110.8	51.1	72.6	231.1^**^		0.94
		08Jiyuan	85.0±11.5	45.2–123.0	60.5	98.9	348.0^**^		0.98
		Combined	73.7±13.0	38.8–123.0	51.5	80.1	405.9^**^	139.8^**^	0.91
RIL	PH	07Zhengzhou	181.6±21.5	105.2–253.4	178.0	222.4	677.1^**^		0.86
		08Zhengzhou	188.2±21.7	110.5–250.0	165.6	185.2	794.9^**^		0.92
		09Xuchang	193.3±20.2	129.0–251.1	179.4	222.8	819.2^**^		0.95
		09Zhengzhou1	207.1±20.3	135.5–258.6	185.0	191.0	904.9^**^		0.94
		09Zhengzhou2	215.6±20.9	137.5–273.0	182.4	190.2	927.2^**^		0.94
		Combined	197.4±24.6	105.2–273.0	178.1	202.3	2565.4^**^	272.7^**^	0.97
	EH	07Zhengzhou	85.0±13.8	46.7–148.0	75.4	102.8	429.5^**^		0.95
		08Zhengzhou	86.7±14.3	53.0–135.0	74.6	83.8	350.7^**^		0.93
		09Xuchang	89.5±15.7	45.3–143.5	73.4	106.3	466.1^**^		0.94
		09Zhengzhou1	101.2±14.6	59.3–149.0	82.8	89.0	486.8^**^		0.95
		09Zhengzhou2	104.4±15.1	53.0–105.5	82.0	92.6	525.4^**^		0.96
		Combined	93.7±16.7	45.3–149.0	77.6	94.9	1623.6^**^	110.0^**^	0.98
GWAS	PH	14Zhengzhou	185.4±29.7	119.4–279.0	–	–	1538.0^**^		0.94
		14Wenxian	206.2±28.3	141.0–293.0	–	–	1499.9^**^		0.97
		15Hainan	163.4±18.1	106.3–214.7	–	–	577.3^**^		0.97
		Combined	185.3±31.2	106.3–293.0	–	–	2619.3^**^	385.3^**^	0.91
	EH	14Zhengzhou	73.5±20.5	24.4–142.0	–	–	743.7^**^		0.94
		14Wenxian	86.6±22.4	27.0–147.6	–	–	947.5^**^		0.97
		15Hainan	57.0±14.4	21.7–96.7	–	–	361.9^**^		0.97
		combined	72.6±22.9	21.7–147.6	–	–	1545.5^**^	179.3^**^	0.93

a The unit of PH and EH is cm.

b SD represents the standard deviation.

c Repeatability for individual environment and heritability for combined environments.

^*^P < 0.05;

** P < 0.01.

### QTL analysis

In the F_2:3_ population, nine QTL were identified for PH and EH (Table 2, Figure S2), and three of them for PH were located on Chr. 1 (bin 1.05/06), Chr. 2 (bin 2.08) and Chr. 5 (bin 5.04/05), and six QTL for EH located on Chr. 1 (bin 1.05), Chr. 2 (bin 2.09/10), Chr. 3 (bin 3.07), Chr. 5 (bin 5.05/06), Chr. 6 (bin 6.04/05), and Chr. 8 (bin 8.06/07). The increasing effect of three QTL for PH and five of the six QTL for EH were originated from the high parent Chang7-2, whereas only one QTL for EH from short parent Zheng58.

**Table 2:** **Quantitative trait loci (QTL) for plant height (PH) and ear height (EH) identified in the F_**2:3**_ population and the recombinant inbred line (RIL) population**.

**Population**	**Trait**	**QTL**	**Bin**	**Marker Interval**	**LOD^a^**	**Additive effects^b^**	**Dominant effects^c^**	**PVE^d^**
F_2:3_	PH	*qPH1-1*	1.05/06	umc1703-umc1590	6.54	−6.78	−0.46	0.11
		*qPH2-1*	2.08	dupssr25-phi090	4.38	−2.4	−3.71	0.07
		*qPH5-1*	5.04/05	umc2302-umc1800	5.76	−4.43	−2.98	0.11
	EH	*qEH1-1*	1.05	umc2112-umc1676	6.86	−4.01	−0.18	0.12
		*qEH2-1*	2.09/10	umc1525-umc2184	3.65	4.65	−1.90	0.06
		*qEH3-1*	3.07	bnlg1605-umc1489	3.00	−2.04	−0.10	0.05
		*qEH5-1*	5.05/06	umc2304-umc1019	4.49	−2.66	−0.95	0.09
		*qEH6-1*	6.04/05	phi38920-bnlg1154	2.03	−2.00	2.54	0.04
		*qEH8-1*	8.06/07	mmc0181-dupssr14	2.18	−0.58	−1.84	0.04
RIL	PH	*qPH2-2*	2.03	umc1776-bnlg1064	5.02	5.37		0.10
		*qPH3-1*	3.04/05	bnlg1452-bnlg1505	6.95	6.56		0.15
		*qPH9-1*	9.02/04	umc1893-umc1492	4.05	4.03		0.06
	EH	*qEH1-2*	1.05/06	umc1676-bnlg1598	6.00	−3.65		0.09
		*qEH2-2*	2.03	umc1776-bnlg1064	2.60	2.50		0.04
		*qEH3-2*	3.04/05	bnlg1452-bnlg1505	11.38	5.43		0.19
		*qEH3-3*	3.08/09	umc2081-bnlg1496	2.51	−2.46		0.04
		*qEH5-2*	5.04/05	umc1990-umc1482	2.43	−2.4		0.04
		*qEH5-3*	5.07/08	bnlg2305-umc1225	2.54	−2.24		0.03
		*qEH6-2*	6.03/04	umc1571-phi031	7.16	−3.92		0.10
		*qEH9-1*	9.02/04	umc1893-umc1492	4.54	3.12		0.07
		*qEH10-1*	10.04	umc1995-umc1053	3.29	2.48		0.04

a Log-likelihood value was calculated by the composite interval mapping method.

b Positive value indicates the increasing effects contributed by parents Zheng58 in the F_2:3_ population and N6 in the RIL population. Negative value indicates the increasing effect from other parents.

c Positive values of the dominance effect indicate that the heterozygotes have higher phenotypic values than the respective means of two homozygotes. Negative values indicate that heterozygotes have lower phenotypic values than the respective means of two homozygotes.

d Explained phenotypic variation.

In the RIL population, 12 QTL were detected for PH and EH: three QTL for PH located on Chr. 2 (bin 2.03), Chr. 3 (bin 3.04/05) and Chr. 9 (bin 9.02/9.04) and nine QTL for EH located on Chr. 1 (bin 1.05/06), Chr. 2 (bin 2.03), Chr. 3 (bin 3.04/05 and bin 3.08/09), Chr.5 (bin 5.04/05 and bin 5.07/08), Chr. 6 (bin 6.03/04), Chr. 9 (bin 9.02/04), and Chr. 10 (bin 10.04) (Table 2, Figure S3). Totally, 21 QTL were identified for PH and EH by two populations, and three common genomic regions (bin 1.05, 5.04/05, and 6.04/05) were detected by both populations.

### GWAS for PH and EH

**Figure 1:**
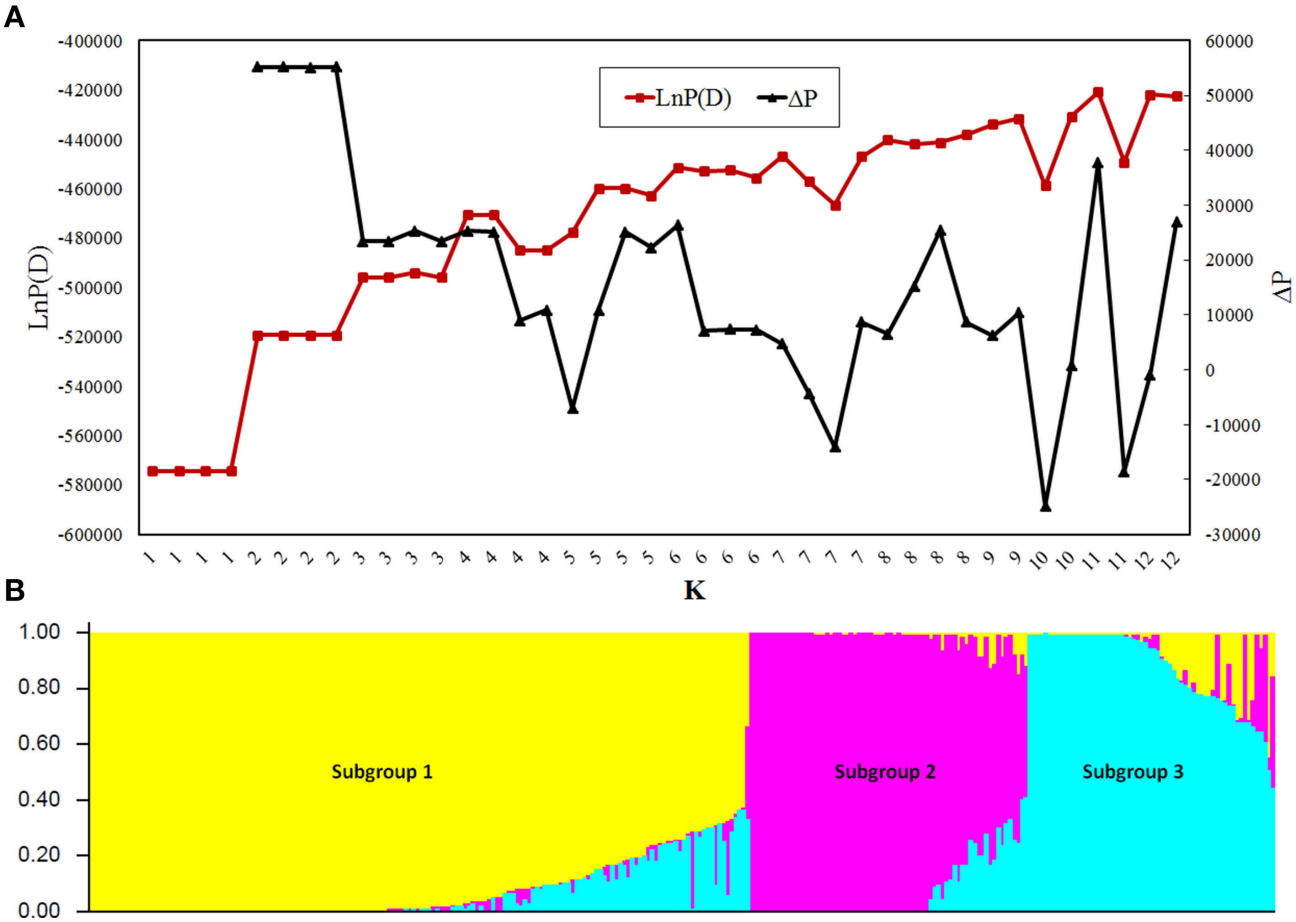
**Population structure of the genome-wide association study (GWAS) panel. (A)** Plot of LnP(D) and an *ad-hoc* statistic ΔP calculated for *K* = 1–12. LnP(D) is the output of STRUCTURE obtained by first computing the natural logarithm of the probability at each step of Markov chain Monte Carlo, ΔP is the difference of LnP(D) between adjacent K. **(B)** Population structure of the GWAS panel at *K* = 3.

Population structure analysis showed that the LnP(D) value was stable between replications when *K* ≤ 3 and became unstable when at *K* > 3 (Figure 1A), indicating that the appropriate *K*-value was 3. At *K* = 3, 98.2% of the GWAS population was divided into Subpopulations 1, 2, and 3, containing 165, 68, and 60 lines, respectively, whereas the rest 2.8% was grouped into a mixed subpopulation (Figure 1B). Subgroup 1 was the most diverse and included tropical lines from CIMMYT; Subgroup 2 included lines from the Tangsipingtou heterotic group and Subgroup 3 included lines from the Reid heterotic group. PCA showed the lines source could separate very well by PC1 and PC2 (Figure S4A), and the first three PCs explained most of the variance (Figure S4B). PCA was in agreement with the results generated by STURCTURE, clearly showing the existence of three subgroups in the GWAS population. Hence, the first three PCs were included in the mixed model for GWAS.

**Figure 2:**
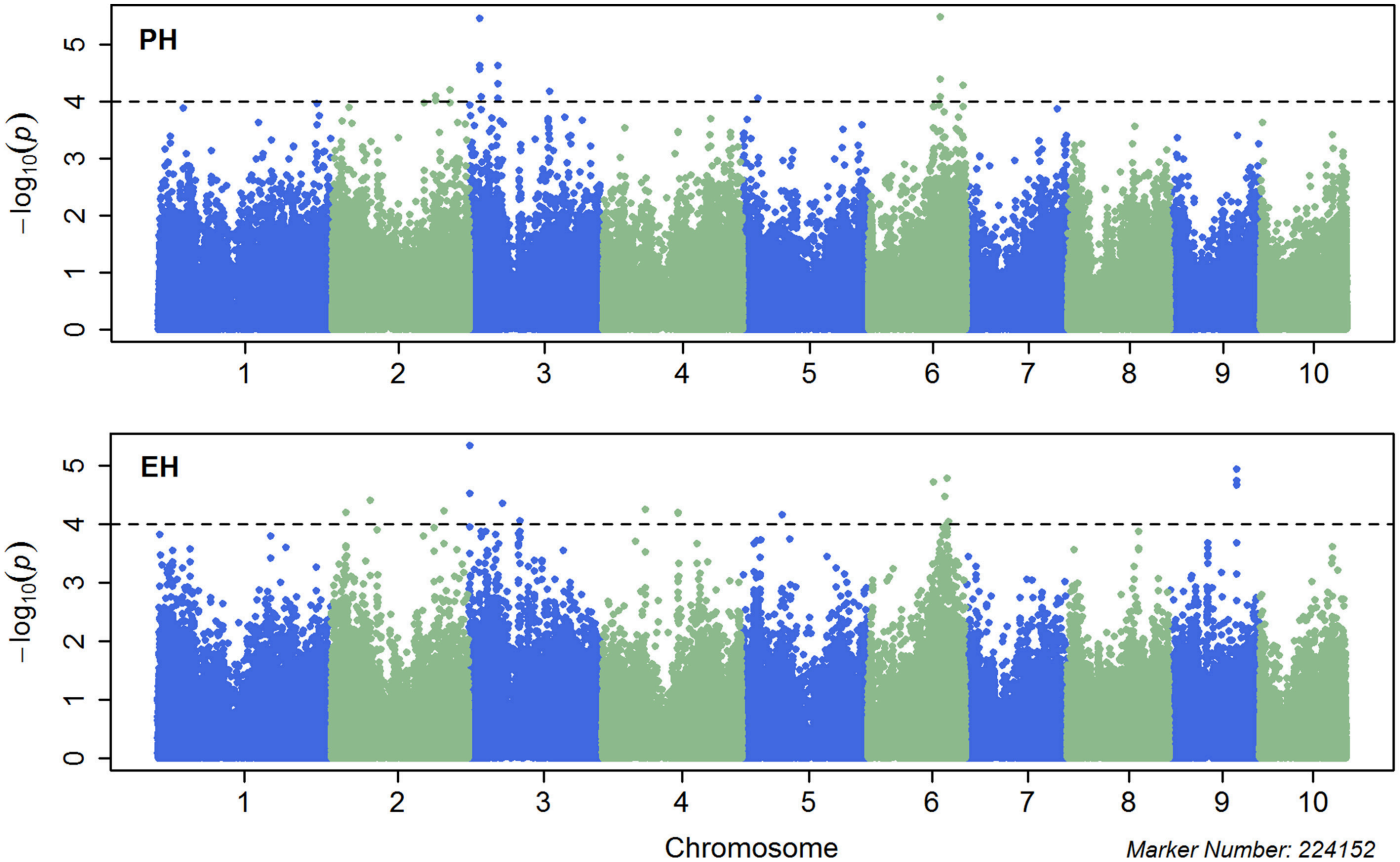
**Manhattan plots for combined genome-wide association study (GWAS) using the mixed linear model (MLM) for plant height (upper plot) and ear height (lower plot)**. Chromosomes and physical positions of single nucleotide polymorphisms (SNPs) on the X-axis and −log10 *p*-value of each SNP derived from the association study on the Y-axis.

Single marker-based GWAS was performed using MLM incorporating both the population structure (first three PCs) and K into the model. A total of 21 significant SNPs for PH and 20 for EH was identified with *p* < 1.0 × 10^−4^ (Figure 2), which explained 6.2–9.3% and 6.2–9.54% of the phenotypic variation for PH and EH, respectively. The most significant SNP was S6_124299082 for PH located on Chr. 6 which explained 9.3% of the phenotypic variation in the GWAS population, whereas the most significant SNP was S3_1976523 for EH located on Chr. 3 which explained 8.8% of the phenotypic variation (Table 3). The quantile-quantile (QQ) plots showed that population structure was well controlled by PCA and K (Figure S5).

**Table 3:** **The significant single nucleotide polymorphisms (SNPs) and their candidate genes associated with plant height (PH) and ear height (EH) identified in this study**.

**Trait**	**SNP**	**Chr**	**Position^a^**	***P*-value**	***R*^2^**	**Candidate gene**	**Annotation**
PH	S2_178613725	2	178613725	8.18E-05	0.064	GRMZM2G116196	Unknown
	S2_178613733	2	178613733	9.82E-05	0.062		
	S2_204259050	2	204259050	6.38E-05	0.074	–	–
	S2_204259051	2	204259051	6.38E-05	0.074		
	S2_204259052	2	204259052	6.38E-05	0.074		
	S2_204259054	2	204259054	6.38E-05	0.074		
	S3_18231963	3	18231963	3.59E-06	0.093	GRMZM5G836162	Mitochondrial substrate carrier family protein
	S3_18232028	3	18232028	2.82E-05	0.076		
	S3_18232030	3	18232030	2.37E-05	0.079		
	S3_20426411	3	20426411	8.37E-05	0.077	GRMZM2G437100	HSP20-like chaperones superfamily protein
	S3_49619525	3	49619525	4.99E-05	0.070	GRMZM2G322186	Pyridoxal phosphate-dependent transferase superfamily protein
	S3_49619564	3	49619564	2.41E-05	0.076		
	S3_49619609	3	49619609	8.91E-05	0.063		
	S3_138737914	3	138737914	6.82E-05	0.083		
	S3_138737941	3	138737941	6.82E-05	0.083	–	–
	S3_138737949	3	138737949	6.82E-05	0.083		
	S5_26139412	5	26139412	9.06E-05	0.073	–	–
	S6_124299082	6	124299082	3.36E-06	0.093	GRMZM2G402538	Acyltransferase with RING/FYVE/PHD-type zinc finger protein
	S6_124300076	6	124300076	4.17E-05	0.077		
	S6_124841703	6	124841703	8.29E-05	0.072	–	–
	S6_164148781	6	164148781	5.25E-05	0.071	GRMZM2G079583	Leucine-rich repeat protein kinase family protein
EH	S2_25387853	2	25387853	6.48E-05	0.062	GRMZM2G031360	Clathrin adaptor complexes medium subunit family protein
	S2_67260246	2	67260246	3.94E-05	0.075	–	–
	S2_195056557	2	195056557	5.99E-05	0.075	GRMZM2G034113	Homeobox associated leucine zipper
	S3_1976523	3	1976523	4.61E-06	0.088	GRMZM2G083749	Helix-loop-helix (bHLH) DNA-binding superfamily protein
	S3_1976526	3	1976526	4.61E-06	0.088		
	S3_2084297	3	2084297	3.01E-05	0.078	GRMZM2G153181	RNA polymerase I-associated factor PAF67
	S3_58714371	3	58714371	4.47E-05	0.068	GRMZM2G378547	Protein kinase superfamily protein
	S3_89609331	3	89609331	8.82E-05	0.063	GRMZM2G503156	AGC (cAMP-dependent, cGMP-dependent and protein kinase C) kinase family protein
	S4_74268672	4	74268672	5.64E-05	0.066	GRMZM2G091503	Signal recognition particle binding
	S4_130433483	4	130433483	6.56E-05	0.062	GRMZM2G127924	Phototropic-responsive NPH3 family protein
	S4_130433496	4	130433496	6.39E-05	0.062		
	S5_69308290	5	69308290	7.01E-05	0.064	GRMZM2G389155	Transducin/WD40 repeat-like superfamily protein
	S6_113668398	6	113668398	1.95E-05	0.073	AC202439.3_FG006	Sodium symporters; urea transmembrane transporters
	S6_132980291	6	132980291	3.46E-05	0.079	–	–
	S6_137828752	6	137828752	1.66E-05	0.079	GRMZM2G170365	RNA-binding (RRM/RBD/RNP motifs) family protein
	S6_139882410	6	139882410	9.22E-05	0.069	GRMZM2G113156	SMAD/FHA domain-containing protein
	S9_117399420	9	117399420	1.82E-05	0.078	GRMZM5G861756	Multimeric translocon complex in the outer envelope membrane
	S9_117399428	9	117399428	1.82E-05	0.078		
	S9_117399471	9	117399471	2.18E-05	0.078		
	S9_117618087	9	117618087	1.16E-05	0.095	–	–

a Position indicates the physical position of B73 RefGen_v2.

Based on the physical position of the significant SNPs in the B73 reference genome, 29 SNPs ware located in 19 unique candidate genes, whereas 12 SNPs in intergenic regions (Table 3). Candidate gene annotation showed that most of the candidate genes function belonged to the kinase family transport and signaling.

### Meta-QTL analysis for PH and EH

**Figure 3:**
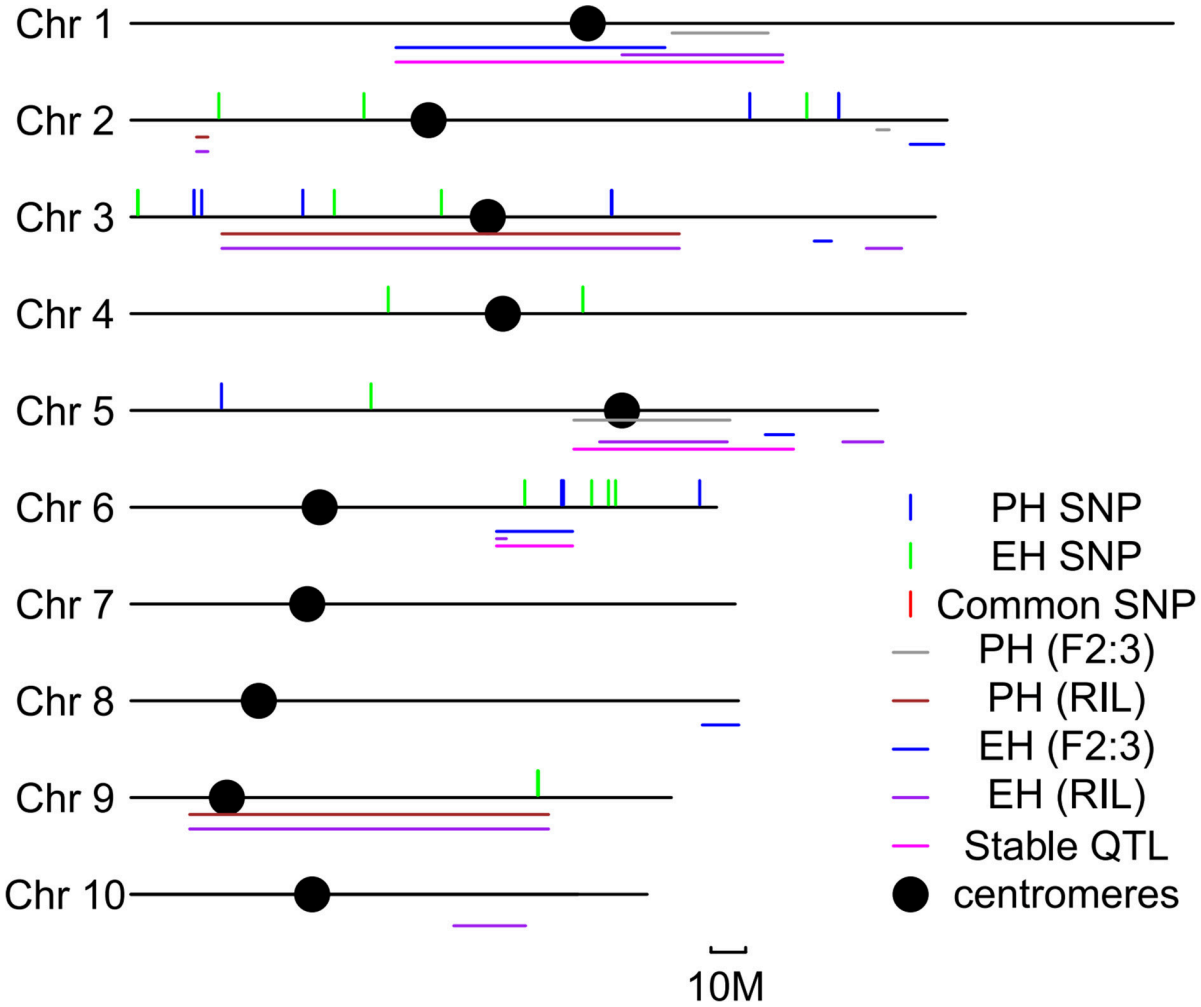
**Meta- quantitative trait locus (QTL) analysis for QTL detected by two bi-parental populations and significant SNPs detected by genome-wide association study (GWAS) in this study**. Colored lines represent different QTL or SNPs detected in this study.

In order to identify the consistent loci for PH and EH, meta-analysis was conducted for both QTL mapping and GWAS results. Six common loci were identified on Chr. 1, Chr. 2, Chr. 3, Chr. 5, Chr. 6, and Chr. 9 (Figure 3). The QTL for PH in bin 1.05 was detected in both the two bi-parental populations, but no SNPs with *p* < 10 × 10^−4^ were identified in the GWAS population; the QTL for EH in bin 6.04 was detected in both the bi-parental populations, and one significant SNP was identified in the GWAS population; the QTL for EH and PH in bin 9.04 was detected in both the bi-parental populations, and one significant SNP was identified in the GWAS population. From meta-QTL analysis, six consistent loci were detected in more than two populations, suggesting that may have stable effects at different genetic backgrounds and environmental conditions.

### QTL verification

**Figure 4:**
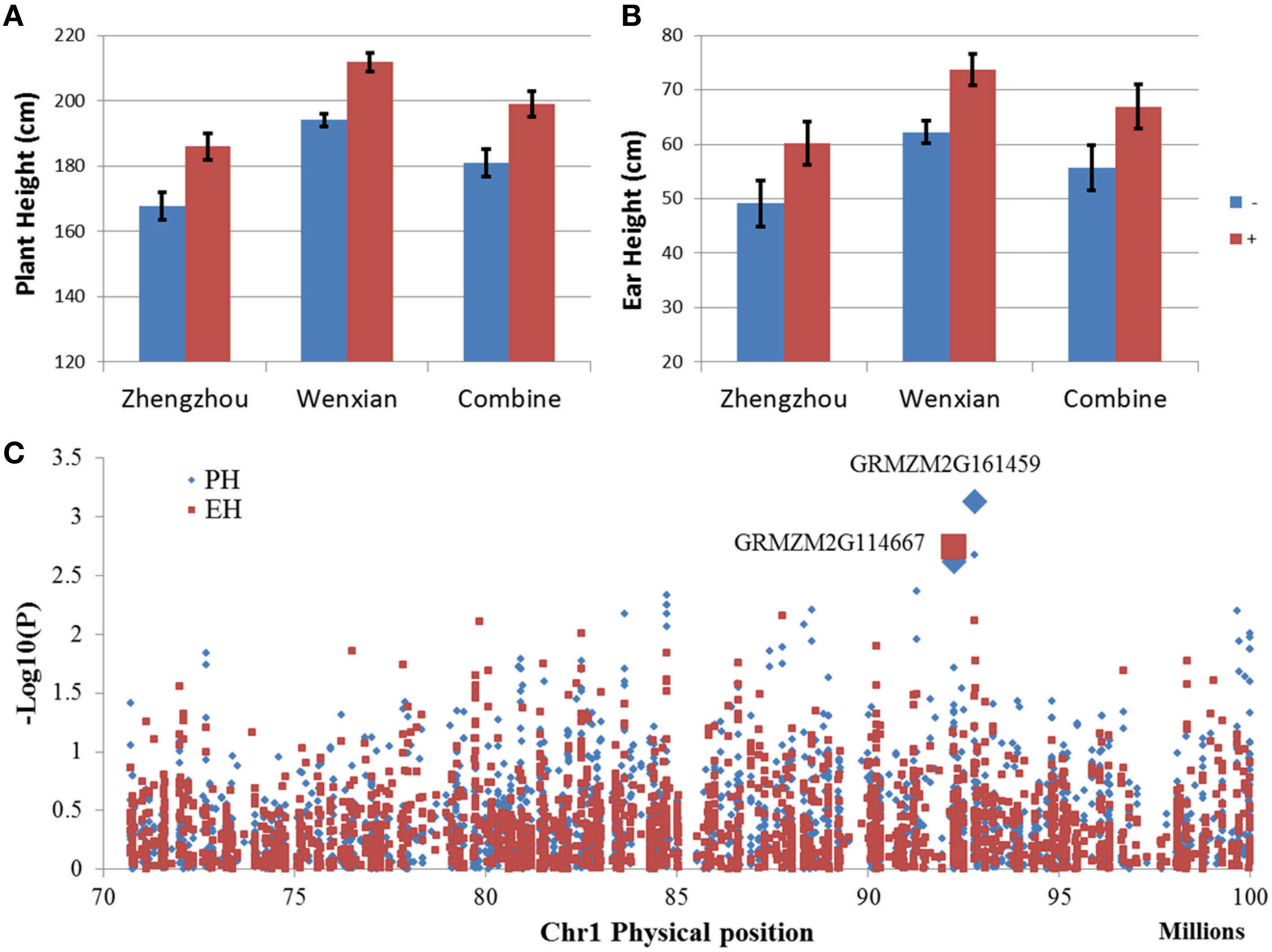
**Quantitative trait locus (QTL) in bin 1.05 was validated using two opposite BC4F3 near isogenic line (NIL) populations and the genome-wide association study (GWAS) result in the QTL region**. **(A,B)** Compare the two BC4F3 NIL populations for plant height **(A)** and ear height **(B)** under different environments. Blue bar indicates the target region of a homozygous allele from the recurrent parent. Red bar indicate the target region of a homozygous allele from the donor parent. **(C)** Manhattan plot for the GWAS result in the QTL region.

The *qPH1-1* for PH in bin 1.05/06 share the same chromosome region of QTL *qEH1-1* for EH, suggesting that they might be the same QTL, controlling both PH and EH. Furthermore, *qPH1-1* had the highest LOD value in the F_2:3_ population. In order to fine mapping this QTL, NIL populations were developed by using marker assistance selection with flanking markers. The phenotype evaluation of NIL populations showed that positive homozygous allele (homozygous from donor parents) could increase PH by 18 cm and EH by 11 cm compared with the negative homozygous allele (Figures 4A,B), verifying QTL mapping results, and indicating that QTL in bin 1.05 could significantly increase PH and EH.

The Manhattan plot of GWAS (Figure 4C) showed that two markers with very low *p*-value, S1_92787084 with *p* = 7.4 × 10^−4^ for PH and *p* = 7.6 × 10^−3^ for EH and S1_92245779 with *p* = 2.4 × 10^−3^ for PH and *p* = 1.8 × 10^−3^ for EH, represented two candidate genes, GRMZM2G161459 and GRMZM2G114667, respectively. GRMZM2G161459 encodes the peptide transporter PTR2, which transports a wide spectrum of dipeptides and is involved in multiple pathways (Chiang et al., 2004; Xia et al., 2008), whereas GRMZM2G114667 encodes the C_2_H_2_ zinc finger family protein which is related to plant development and is required for the transcription of a number of genes coding proteins with stress-protective functions (Görner et al., 1998; Iuchi, 2001). Additionally, GRMZM2G114667 is highly expressed in the internode of the plant stem and may relate to the development of internodes. Compare with GRMZM2G161459, GRMZM2G114667 has higher possibility to be the candidate gene of *qPH1-1*, because of it is highly expressed in the internode, has a similar high effect on both PH and EH, and is highly related to plant development.

## Discussion

### Heredity of height in maize

Height is one of the most important agronomic traits and the most heritable trait in maize. However, the underlying genetic mechanisms of natural variation remain unclear. In the present study, the normal distribution and high heritability of PH and EH variation were detected in both the bi-parental and GWAS populations, suggesting that height in maize is a highly polygenic trait. The large number of markers with small effects that detected in GWAS confirmed that height in modern maize inbred lines is a polygenic trait controlled by multiple genes with low effect. These results were in agreement with previous studies (Peiffer et al., 2013, 2014).

### Relationship between PH and EH

PH and EH are significantly correlated in maize breeding populations, thus some QTL for these traits are mapped in the same genomic regions due to pleiotropic effects (Tang et al., 2006). In the present study, QTL for both PH and EH were located in the same genomic region such as *qPH1-1* for PH and *qEH1-1* for EH (bin 1.05/06) in the F_2:3_ population as well as *qPH3-1* for EH and *qEH3-2* for EH (bin 3.04/05) in the RIL population. However, some QTL were only mapped for one of the two traits such as *qEH6-2* for EH in the RIL population. These results suggested that the genetic mechanism of PH and EH may be similar, but not identical.

### Genome wide association study

GWAS is a powerful approach for exploring the molecular basis of phenotypic variations in plants, and the population is a foundation for GWAS. There were several GWAS populations have been reported for PH and EH research (Weng et al., 2011; Yang et al., 2011; Peiffer et al., 2014). The different population have different features, some populations used global diversity of lines which focus on global variation analysis (Yang et al., 2011; Peiffer et al., 2014), and others may only use region germplasm which focus on region variation analysis. For example, Weng et al. (2011) used a GWAS population contained Chinese elite lines identified a major QTL for height on chromosome 5, which played an important role in Chinese maize breeding programs. In the present study, the GWAS population mainly contained Chinese elite inbred lines and some tropical inbred lines from CIMMYT, it represented most of the diversity of Chinese and tropical germplasm which were the important germplasm in Chinese breeding programs. The Chinese and tropical germplasm were used for GWAS analysis, in order to reveal the genetic basis of height nature variation in these important germplasm in breeding programs. The further analysis on diversity comparison between present GWAS population and previous reported population was profitable to understand the different significant markers detected in different populations and may facilitate a better understanding of the underlying mechanism of height in maize.

GWAS has been widely used for functional gene discovery that has yielded a large number of associations between markers and various complex traits (Visscher et al., 2012; Li et al., 2016). However, some markers with high significant *p*-value may not be genuine whereas others with moderate or even low significant *p*-values can be considered genuine based on the biological trait (Panagiotou and Ioannidis, 2012). The selection of the most suitable threshold for GWAS using statistical approaches, such as the Bonferroni correction (Bland and Altman, 1995) or the false discovery rate (Benjamini and Hochberg, 1995), is important for the identification of genuine markers for target traits. A fixed cut-off value based on the number of markers and traits, such as *p* < 10^−5^ or *p* < 10^−4^, provides flexibility and is commonly used in GWAS. In the present study, no SNPs passed the Bonferroni correction threshold, probably because height is a complex traits in modern maize inbred lines controlled by multiple genes with low effect (Peiffer et al., 2014). Therefore, to identify the top SNP associated with PH and EH, we choose a fixed cut-off threshold of *p* < 10^−4^.

### Comparison with QTL identified in previous studies

Previous studies have shown that common QTL can be detected in different environments and populations (Beavis et al., 1991; Chen et al., 2012). In the present study, we detected nine QTL for PH and EH in the F_2:3_ families population and 12 QTL for PH and EH in the RIL populations. Of these, three “stable QTL” (detected in both bi-parental populations) were identified for PH and EH on Chr. 1, Chr. 5, and Chr. 6 and also reported in previous studies (Cai et al., 2012; Park et al., 2013; Sa et al., 2014; Wei et al., 2015). These results indicated that those three loci were stable QTL for PH and EH in modern maize inbred lines.

### Candidate genes for height in maize

The QTL for EH on Chr. 6 (bin 6.04/05) could explain 4% of the phenotypic variation in the F_2:3_ population and 10% of the phenotypic variation in the RIL population. However, no QTL was detected in this genomic region for PH in these two populations. The associated SNP S6_113668398 had the lowest *p*-value (*p* = 1.95 × 10^−5^) and explained 7.3% of the phenotypic variation in the GWAS population for EH. Candidate gene analysis showed that S6_113668398 was located in AC202439.3_FG006, which encodes a urea transmembrane transporter. The homologous genes in *Arabidopsis* (AtDUR3), and rice (LOC_Os10g42960.1) also encode transporters that play an important role in the uptake and use of urea (Kojima et al., 2007; Wang et al., 2008). Urea is the main source of nitrogen in modern crop production, because of its low cost and easy absorption by the plants, and provides 50% for the total weight of nitrogen fertilizer worldwide (Kojima et al., 2007; Wang et al., 2008). The efficient use of the nitrogen by the plant increases its height (Lafitte and Edmeades, 1994). Therefore, this gene could be considered an important candidate gene for maize height, and further functional verification might help to better understand the underlying genetic mechanism.

The QTL for PH and EH on Chr. 3 could explain 15 and 19% of the phenotypic variance for PH and EH, respectively, in the RIL population. An associated SNP with small *p*-value was detected for PH located in the annotated gene GRMZM2G322186. GRMZM2G322186 encodes a pyridoxal phosphate-dependent transferase and also has multiple catalytic functions in various organisms (Percudani and Peracchi, 2003). The protein level of GRMZM2G322186 was highly different between two parental lines BT-1 and N6 of the RIL population, and the N6 showed similar protein sequence with reference genome B73 (Figure S6). Expression analysis showed that GRMZM2G322186 has a high expression level in all the developmental stages of maize, indicating its importance in plant development (Winter et al., 2007; Sekhon et al., 2011). These results suggested that GRMZM2G322186 was the most possible candidate gene for the QTL of PH on Chr. 3.

## Conclusions

In this study, two bi-parental populations and one GWAS population were used to identify and map QTL for PH and EH. A total of 21 QTL for PH and EH was detected by QTL mapping and 41 SNPs were identified by GWAS. A QTL on Chr. 1 was verified in NILs and indicated that a C_2_H_2_ zinc finger family protein might be the candidate gene for PH. One candidate gene on Chr. 6 (AC202439.3_FG006), encoding a urea transmembrane transporter, was considered as the candidate gene for EH. The candidate genes for other stable QTL were also discussed. These results confirmed that nature variation of maize height is strongly controlled by multiple genes with low effect and the QTL and candidate genes identified in this study could help to better understand the genetic basis of PH and EH in maize.

## Author contributions

JW, JC, JD, and XZ designed, led, and coordinated the overall study. XL, ZZ, YW, BZ, RW, JM, SW, and XZ perform the field experiment. ZZ, XL, and JC carried out the analysis. JC, ZZ, XL, and JW wrote the manuscript.

## Funding

The work was supported by National Natural Science Foundation of China (Grant No. 31271307).

### Conflict of interest statement

The authors declare that the research was conducted in the absence of any commercial or financial relationships that could be construed as a potential conflict of interest.

## Supplementary material

Figure S1 **Phenotypic distribution of plant height (PH) and ear height (EH) in the F_**2:3**_ population, the recombinant inbred line (RIL) population, and the genome-wide association study (GWAS) population**. The density line is shown in black.

Figure S2 **Quantitative trait loci (QTL) detected for plant height (PH) and ear height (EH) in the F_2:3_ population**.

Figure S3 **Quantitative trait loci (QTL) detected for plant height (PH) and ear height (EH) in the recombinant inbred line (RIL) population**.

Figure S4 **Principal components analysis (PCA) for genome-wide association study population. (A)** Plot of the first two PCs separated for the heterotic group; **(B)** Eigenvalue of the first 15 PCs.

Figure S5 **Quantile-quantile plot of genome-wide association study (GWAS) for plant height (PH) and ear height (EH)**.

Figure S6 **Comparison of the protein sequence of the candidate gene GRMZM2G322186 between the two parental lines (BT-1 and N6) of the recombinant inbred line (RIL) population and the B73 reference genome**.

File S1 **R code for meta-quantitative trait locus (QTL) analysis**.
