# Supplementary figures and images for: Combined Linkage and Association Mapping Reveals QTL and Candidate Genes for Plant and Ear Height in Maize
Source: Front Plant Sci. 2016 Jun 15;7:833. doi: 10.3389/fpls.2016.00833 (PMC4908132; doi:10.3389/fpls.2016.00833)

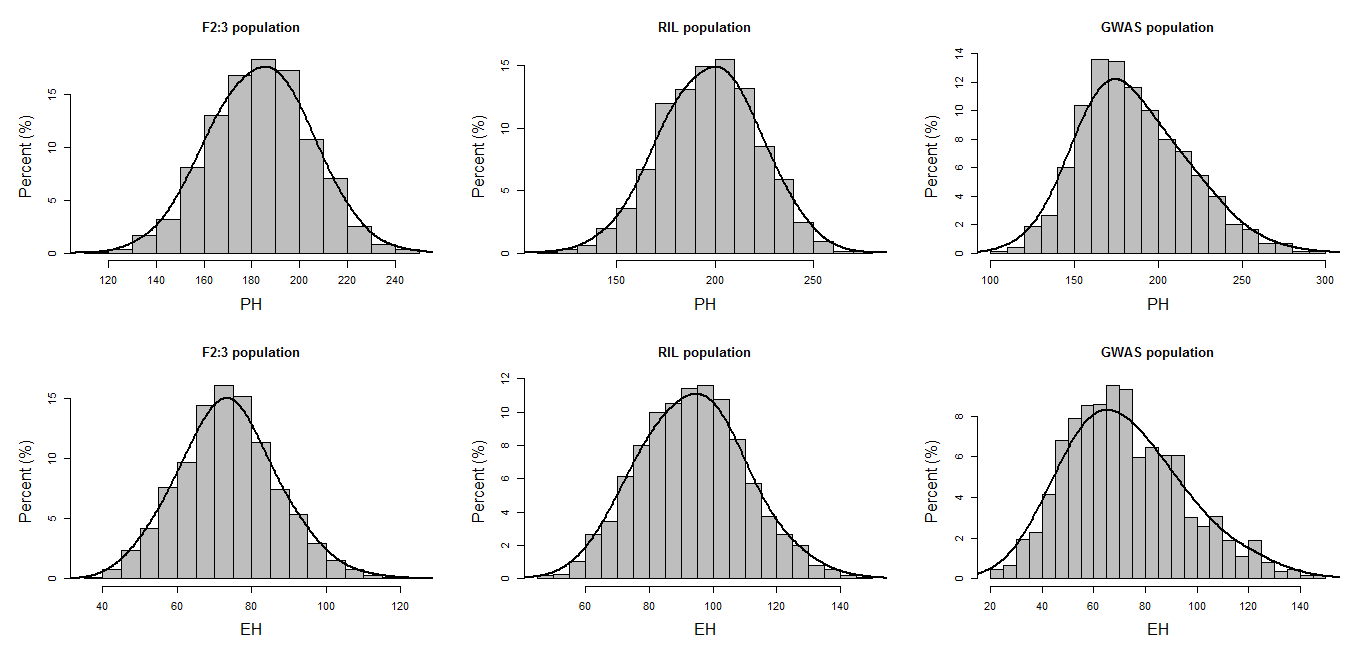

Supplement: Figure S1 — Phenotypic distribution of plant height (PH) and ear height (EH) in the F2:3 population, the recombinant inbred line (RIL) population, and the genome-wide association study (GWAS) population. The density line is shown in black. [file Image1.TIF]

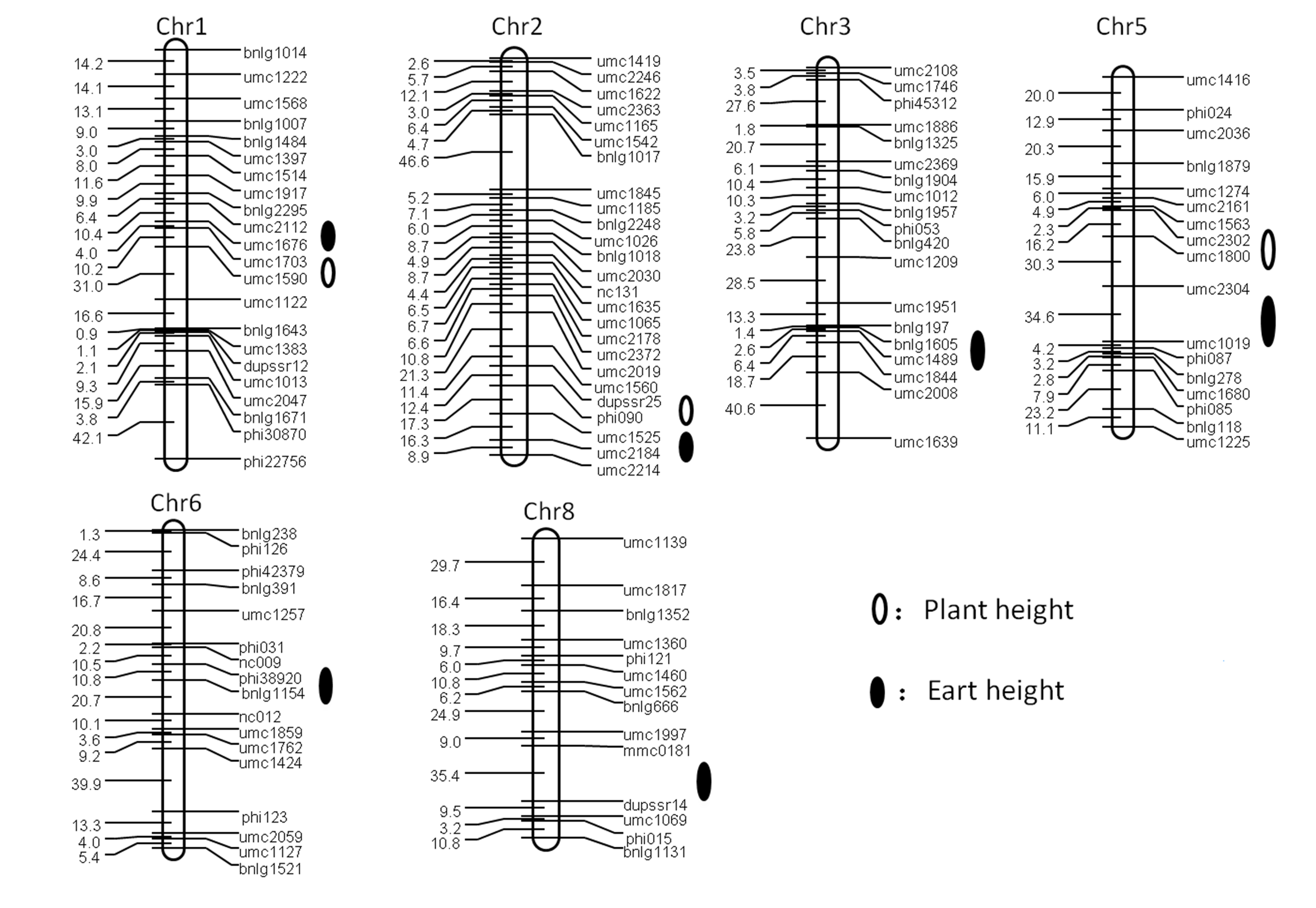

Supplement: Figure S2 — Quantitative trait loci (QTL) detected for plant height (PH) and ear height (EH) in the F2:3 population. [file Image2.TIF]

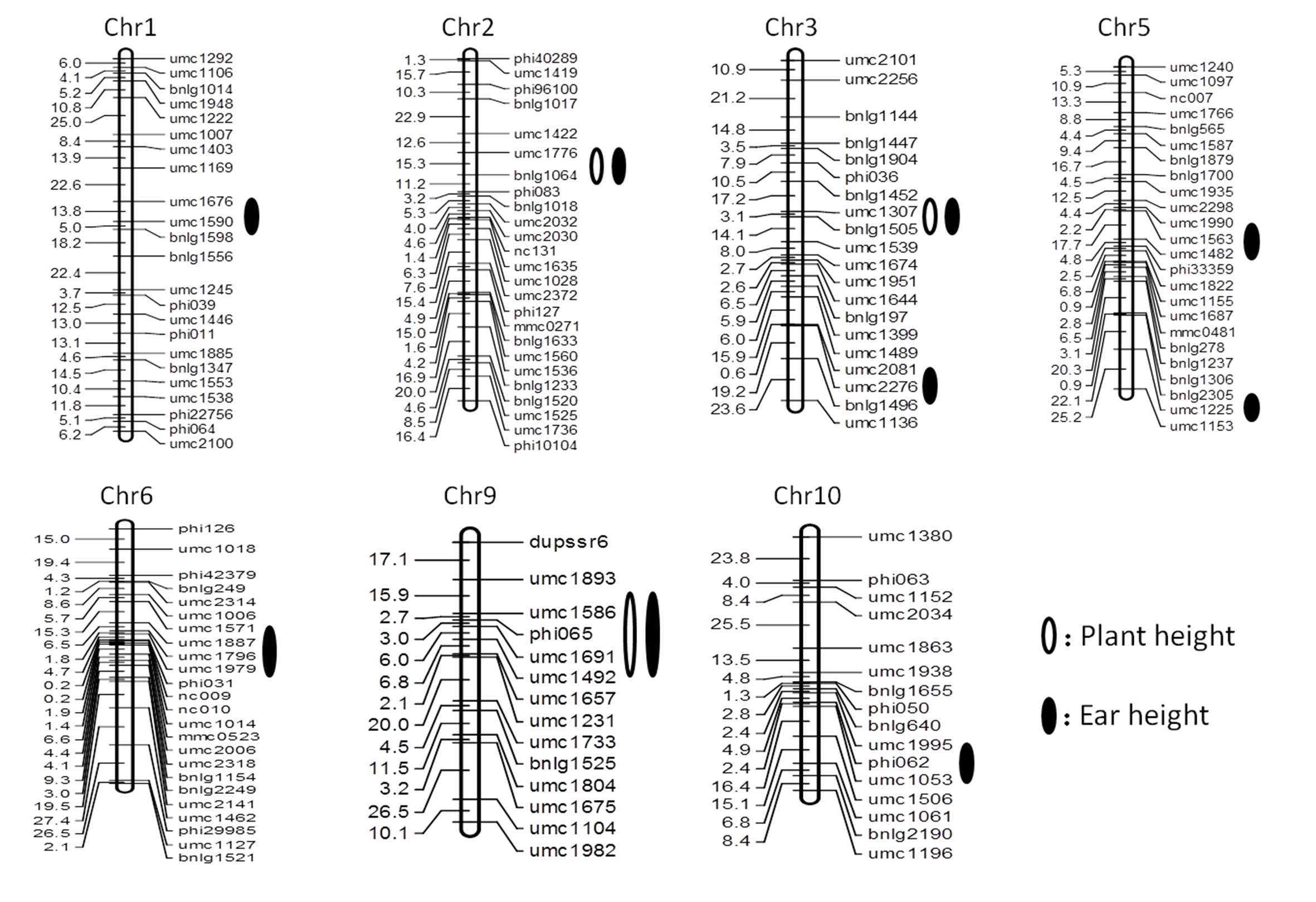

Supplement: Figure S3 — Quantitative trait loci (QTL) detected for plant height (PH) and ear height (EH) in the recombinant inbred line (RIL) population. [file Image3.TIF]

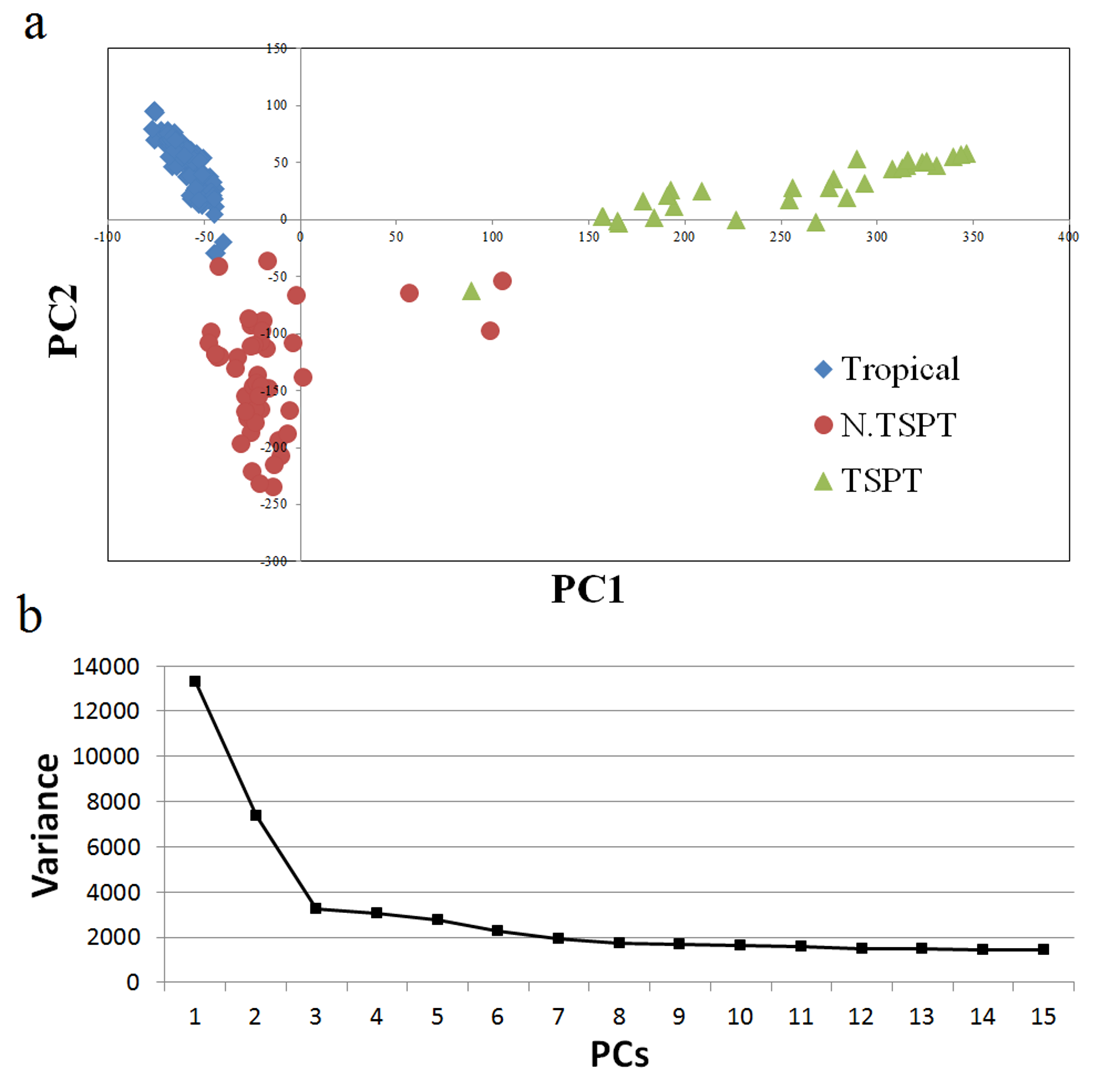

Supplement: Figure S4 — Principal components analysis (PCA) for genome-wide association study population. (A) Plot of the first two PCs separated for the heterotic group; (B) Eigenvalue of the first 15 PCs. [file Image4.TIF]

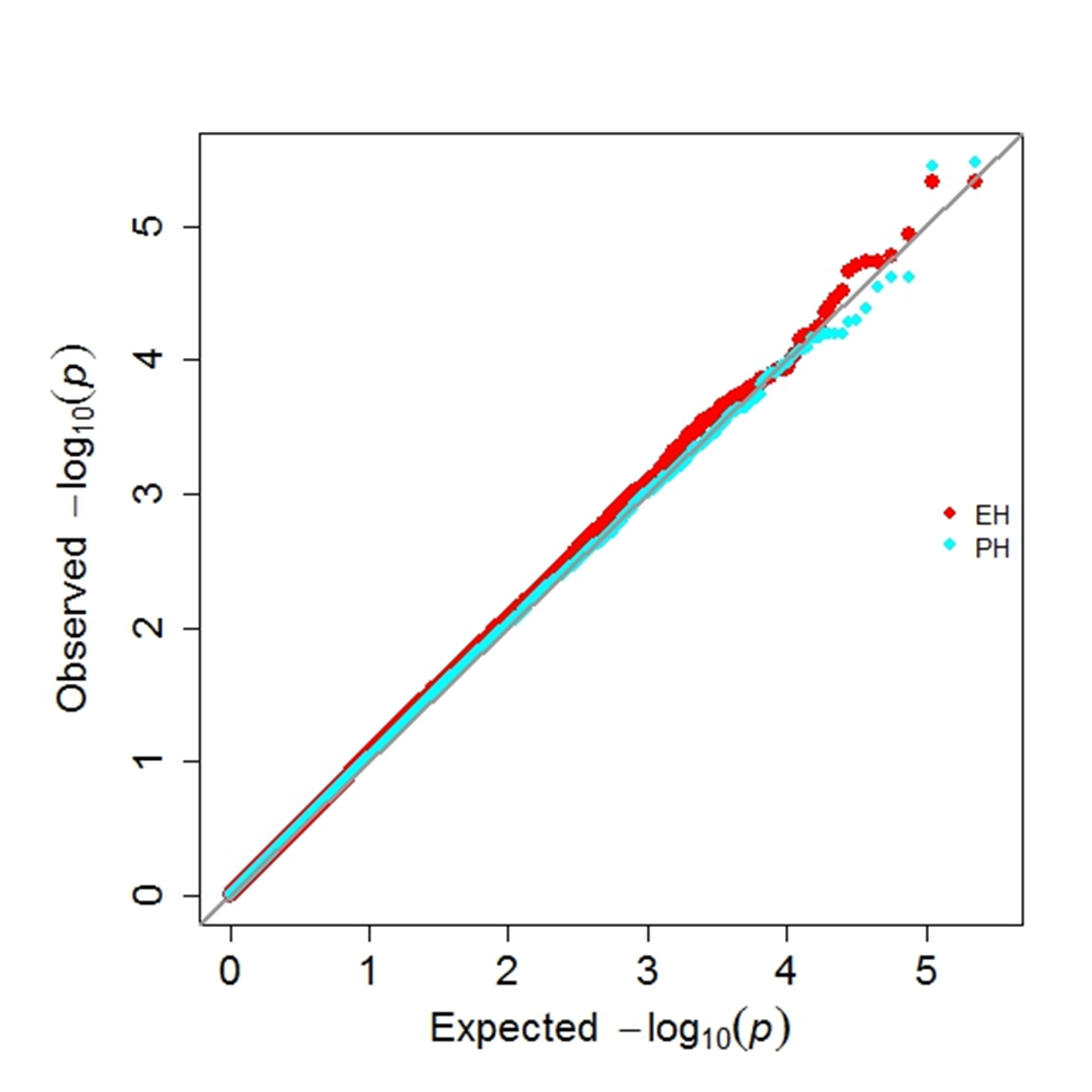

Supplement: Figure S5 — Quantile-quantile plot of genome-wide association study (GWAS) for plant height (PH) and ear height (EH). [file Image5.TIF]

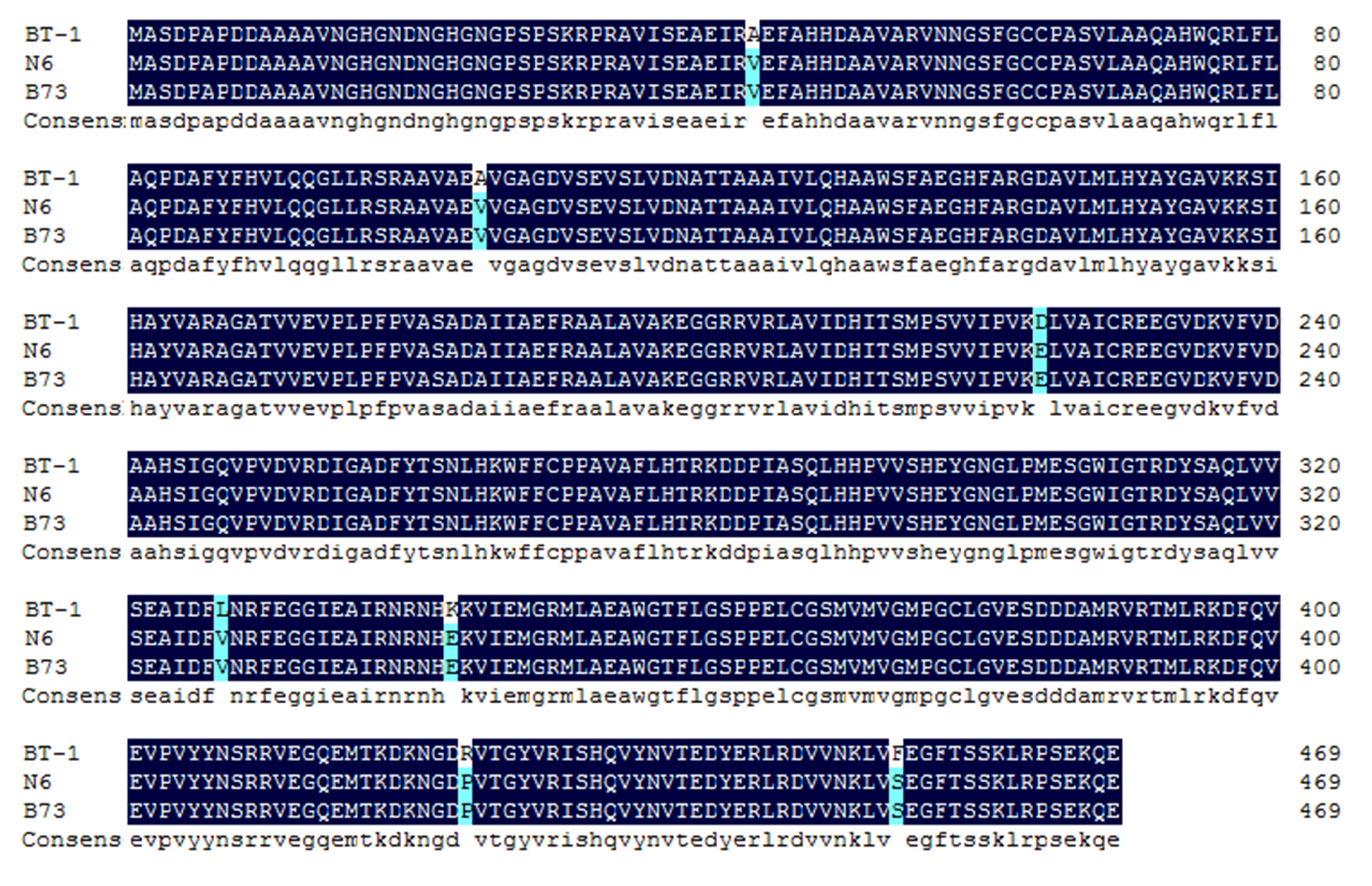

Supplement: Figure S6 — Comparison of the protein sequence of the candidate gene GRMZM2G322186 between the two parental lines (BT-1 and N6) of the recombinant inbred line (RIL) population and the B73 reference genome. [file Image6.TIF]
